# Anxiety symptoms and coping strategies among high school students in Vietnam after COVID-19 pandemic: a mixed-method evaluation

**DOI:** 10.3389/fpubh.2024.1232856

**Published:** 2024-02-16

**Authors:** Pham Thi Thu Hoa, Do Thi Trang, Nguyen Thi Lien, Ngo Anh Vinh

**Affiliations:** ^1^Faculty of Psychology, University of Social Sciences and Humanities, Vietnam National University, Hanoi, Vietnam; ^2^Bình Minh Center for Education Psychology Research and Application, Hanoi, Vietnam; ^3^Faculty of Educational Sciences, University of Education, Vietnam National University, Hanoi, Vietnam; ^4^Department of Adolescent Health, Vietnam National Children's Hospital, Hanoi, Vietnam

**Keywords:** anxiety disorder, coping strategies, adolescent, high school, COVID-19 pandemic

## Abstract

**Introduction:**

The objective of the current study was to examine the rate of high school students at risk of anxiety disorder during the COVID-19 pandemic in Vietnam, as well as the coping strategies utilized within this demographic.

**Methods:**

An evaluation was conducted through the utilization of mixed methods, consisting of a combination of a cross-sectional study and in-depth interviews. In this study, a sample of 3,910 students from 13 high schools in Hanoi, Vietnam were selected for participation. The measurement of symptoms of anxiety disorder was conducted through the application of the seven-item General Anxiety Disorder (GAD-7) scale. To comprehend the underlying causes of anxiety and the various coping mechanisms employed, in-depth interviews were conducted.

**Results:**

The findings indicate a prevalence rate of anxiety disorder symptoms among students at 40.6% The prevalence rates of mild, moderate, and severe anxiety symptoms were found to be 23.9%, 10.9%, and 5.8%, respectively. In-depth interviews uncovered multiple sources of anxiety experienced by high school students, namely their academic performance, social interactions, prejudicial attitudes from their social circle, and familial expectations. Numerous coping strategies were then documented.

**Discussion:**

The current investigation ascertained that there exists a moderate level of anxiety amongst high school students in Hanoi, Vietnam during the COVID-19 outbreak. Furthermore, this study configured potential indicators to identify vulnerable individuals and further suggests the development of targeted interventions.

## 1 Introduction

Adolescence is a distinct period of maturation marked by significant physiological, psychological, and social transformations, rendering these individuals potentially more susceptible to deleterious impacts of stressful circumstances (1, 2). According to a meta-analysis comprising 136 studies conducted on diverse populations impacted by the COVID-19 pandemic, a minimum of 15–16% of the general populace suffered from symptoms related to anxiety or depression (3). During the COVID-19 pandemic, while various feelings such as the fear of contracting an infection, grief over loss, and a sense of overpowering uncertainty were collectively felt by individuals of varying ages, the extensive disruption of education had a significant impact on the mental wellbeing of children and adolescents.

Prior to the emergence of the current pandemic, there were well-documented and significant instances of increased anxiety, depression, substance abuse, and other related mental health challenges experienced by adolescents who faced high academic and societal pressures to succeed (4). The adoption of remote learning, implementation of social gathering limitations, alteration or discontinuation of sports or clubs, and the suspension of in-school activities and events pose significant obstacles to the intellectual and communal development of juveniles. The perturbation in educational processes and the resultant seclusion, coupled with the absence of guidance from instructors and classmates, not only impedes scholarship but may also intensify the apprehension experienced by adolescents about their academic pursuits and professional ambitions (5, 6). A prior study among adolescent students found that students who received virtual education had more mentally unhealthy days, more depressive symptoms, and a tendency to think about suicide compared to peers with different forms of education (7). The offering of social support by peers and educators is considered a crucial element in safeguarding the social-emotional wellbeing of adolescents, especially during periods of stress. Furthermore, the provision of such support could potentially contribute to the sustained academic engagement and motivation of this particular group (8). Students who received virtual instruction reported experiencing a higher number of mentally unhealthy days, more persistent symptoms of depression, and a greater likelihood of contemplating suicide compared to their peers who were instructed through different modalities (7).

In Vietnam, during the phase of adolescence, individuals undergo a challenging transition, rendering them highly susceptible to the negative impacts posed by the COVID-19 pandemic. A previous population-wide study showed that among university students, 16.2% suffered from anxiety during the COVID-19 pandemic (9). In response to the COVID-19 pandemic, educational institutions nationwide implemented temporary closures and directed students to remain in their respective homes. Adolescents may experience significant psychological impacts due to decreased social interaction, academic challenges, fear of illness, substantial alterations in their daily routine, and feelings of boredom. It has been observed that they exhibited heightened vulnerability to the psychological ramifications of the epidemic, with a corresponding lack of efficacy in coping with their psychological distress. Nevertheless, a limited number of investigations have been undertaken to explore the psychological wellbeing challenges encountered by this demographic amidst the COVID-19 outbreak in Vietnam. The objective of the current study was to examine the rate of high school students at risk of anxiety disorder during the COVID-19 pandemic in Vietnam, as well as the coping strategies utilized within this demographic.

## 2 Materials and methods

### 2.1 Study settings and participants

A cross-sectional study was undertaken among high school students in Hanoi, Vietnam, from October to November 2021, in response to the lockdown imposed in Hanoi due to the COVID-19 pandemic. During this stage, students are required to engage in remote learning from home. The study required participants to conform in full to the following stipulations: (1) an age range from 14 to 17; (2) residency in Hanoi, Vietnam coupled with enrollment in a high school chosen for its inclusion in the study; and (3) explicit consent to partake in the research. The research methodology employed a multi-stage sampling technique. Initially, a list of high schools in Hanoi was compiled and stratified according to geographical location (rural/urban) and institutional classification (private/public), resulting in four possible groups (rural/private, rural/public, urban/private, and urban/public). Thirteen high schools were chosen at random in order to ensure that each group would have at least three high schools. The selected schools are independent of the school's performance and are typical options for high school students. The entirety of eligible students encompassed within these educational institutions were chosen. A cohort of 3,910 pupils enrolled from 13 secondary educational establishments in Hanoi. This study was approved by the institutional review board of the Vietnam National University.

### 2.2 Measurement and instrument

The current study employed the Google Form as the framework to construct an electronic survey. This methodology offers various benefits, including cost-effectiveness, expeditiousness, ease of use, and the ability to attain a sample of a wide geographical coverage. Each survey is expected to require approximately 10 to 15 min for completion. The present study gathers sociodemographic data, anxiety disorder data, and related information about the COVID-19 pandemic via the utilization of a structured questionnaire. At the outset, the questionnaire underwent a pilot test with a cohort of five adolescent individuals to substantiate that the ethnically diverse characteristics of the instruments persisted unaltered after their translation into the Vietnamese language. Subsequently, the revised questionnaire was disseminated through the online survey platform. Before the commencement of data collection, the survey system underwent rigorous testing to verify the accuracy of the questions and to ascertain that no technical complications emerged.

The study participants provided data on their socio-demographic characteristics, including gender, educational institution category, level of schooling, and geographic location. In the present study, the Generalized Anxiety Disorder 7-Item (GAD-7) Scale was employed as an assessment tool to quantify the rate of at-risk anxiety disorders among adolescent participants enrolled in high school education (10, 11). The respondents assessed the frequency of their occurrence of symptoms associated with generalized anxiety disorder, such as emotional irritability or annoyance, using a rating scale ranging from 0 to 3 representing the absence of symptoms and daily manifestation over the last fourteen consecutive days, respectively. A classification framework was employed to delineate the levels of anxiety among the students. Specifically, the classification system comprised four distinct levels, namely: no anxiety, mild anxiety, moderate anxiety and severe anxiety which were based on a scoring range of 0–4, 5–9, 10–14 and 15–21, respectively. The psychometric properties of GAD-7 scores (which have a potential range of 0–21) were found to be satisfactory with adolescent populations. The Cronbach's alpha coefficient attained a value of 0.916.

In qualitative analysis, 20 high school students were selected at random from the available database and approached through telephone communication to obtain informed consent for a recorded interview. A convenient date and time were then scheduled for interviewing telephonic means. Participants were recruited until a point of saturation was achieved about the responses proffered by the participants.

### 2.3 Statistical analysis

SPSS version 20.0 was used to analyze the data. The significance level was set at 5% which is equivalent to a *p* ≤ 0.05. In a typical descriptive statistical study, the mean and standard deviation for quantitative data, as well as frequency and percentage for qualitative variables, were employed. The internal consistency reliability was examined by calculating Cronbach's alpha, with an alpha value of 0.7 or above being considered acceptable.

The analysis and reporting of qualitative data entail the consolidation of all responses obtained from participants into a singular dataset. The present study employed qualitative content analysis to transcribe and analyze the audio-recorded responses provided by students. Initially, the procedure involved a comprehensive review of the responses contained within the scripts, to gain a thorough understanding of their substance. Following this stage, meaningful segments were subsequently identified as discrete units of analysis. The semantic units were subsequently encoded under their subject matter, and these encodings were subsequently aggregated into taxonomic classes. The authors of the study undertook the tasks of data summarization, coding, and category development through the application of thematic analysis.

## 3 Results

Table 1 shows that, among 3,910 students, the majority of them were female (51.4%), studying in urban areas (52.5%). Most of the students studied in public schools (52.9%) and grade 12 (35.9%).

**Table 1:** Demographic characteristics of respondents (*n* = 3,910).

**Characteristics**		**Freq**.	**Percent (%)**
Gender	Male	1,851	47.3%
	Female	2,009	51.4%
	Others	50	1.3%
Location	Urban	2,052	52.5%
	Rural	1,858	47.5%
Type of schools	Public	2,069	52.9%
	Private	1,841	47.1%
Grade	Grade 10	1,381	35.3%
	Grade 11	1,127	28.8%
	Grade 12	1,402	35.9%

Table 2 shows the profile of GAD-7 instrument among high school students. The most common symptom was “Becoming easily annoyed or irritable” (mean score = 1.01), followed by “Worrying too much about different things” (mean score = 0.96) and “Feeling nervous, anxious, or on edge” (mean score = 0.89). Overall, the rate of high school students who were at risk of anxiety disorder was 40.6%. The proportion of mild, moderate and severe anxiety symptoms accounted for 23.9%, 10.9% and 5.8%, respectively.

**Table 2:** Profile of general anxiety disorder (GAD-7) instrument.

**TT**	**Items**	**Level**								**Mean**
		**Not at all**		**Several days**		**More than half the days**		**Nearly every day**		
		***N***	**%**	***N***	**%**	***N***	**%**	***N***	**%**	
1	Feeling nervous, anxious, or on edge	1,574	40.3	1,658	42.9	359	9.2	319	8.2	0.89
2	Not being able to stop or control worrying	2,320	59.3	1,072	27.4	303	7.7	215	5.5	0.85
3	Worrying too much about different things	2,029	51.9	1,117	28.6	416	10.6	348	8.9	0.96
4	Trouble relaxing	2,111	54.0	1,187	30.4	356	9.1	256	6.5	0.89
5	Being so restless that it is hard to sit still	2,729	69.8	833	21.3	205	5.2	143	3.7	0.75
6	Becoming easily annoyed or irritable	1,754	44.9	1,247	31.9	453	11.6	456	11.7	1.01
7	Feeling afraid, as if something awful might happen	2,637	67.4	824	21.1	244	6.2	205	5.2	0.83

Table 3 depicts that there were significant differences in the GAD-7 score regarding gender, location and grade (*p* < 0.05).

**Table 3:** Anxiety disorder scores according to different demographic characteristics.

**Characteristics**		**Freq**.	**Percent (%)**	**Mean**	**SD**	***p*-value**
Gender	Male	1,851	47.3%	1.50	0.83	< 0.01
	Female	2,009	51.4%	1.74	0.92	
Location	Urban	2,052	52.5%	1.68	0.92	< 0.01
	Rural	1,858	47.5%	1.59	0.86	
Type of schools	Public	2,069	52.9%	1.61	0.88	0.08
	Private	1,841	47.1%	1.66	0.91	
Grade	Grade 10	1,381	35.3%	1.53	0.85	< 0.01
	Grade 11	1,127	28.8%	1.64	0.87	
	Grade 12	1,402	35.9%	1.72	0.94	

### 3.1 Qualitative analysis regarding challenges, causes and coping strategies among high school students during the COVID-19 pandemic lockdown

There were many causes of anxiety disorders among high school students in Hanoi, in which work and study were the leading causes of anxiety disorders among students.

“*Last time of COVID-19, I had just entered 10th grade, so I was quite subjective and busy playing, neglecting my studies plus studying online, the school was less tightly managed than going to face-to-face classes, so there were a few subjects that I didn't like. as if it was completely lost. Now I'm in grade 11 and go back to school directly, losing my roots makes it quite difficult for me to study and my study results are very bad compared to the common ground, I have been under a lot of pressure because of this. that. Because of that, my mother often has harsh words that make me more confused”* (A male student in a public school).

The second reason was social relationships. Students' social relationships often revolved around friends and teachers in school. Students often feel inferior to their friends and feel anxious when they were better than them. This feeling could be considered as a positive factor to motivate students to strive, try to overcome difficulties and overcome themselves. Instead of utilizing this anxiety as a motivator for self-improvement, some students perceive this anxiety as causing them to lose sleep, consequently leading to this anxiety becoming a pathological condition or a negative factor, rather than a positive one (12). Many students experienced stress and anxiety just because of the standards they set for themselves.

“*When I studied online, my relationship with my best friend was really bad. We rarely talk to each other, even for a week we don't talk to each other. Even after going back to school, I was isolated and slandered by my classmates”* (A female student in a rural high school).

In addition, some students had conflicts in their relationship with teachers, even some were very inhibited when it came to a certain teacher's class time. “*Since switching to online learning, my teacher curses students a lot. That's why I feel so pressured every time I take her classes*.”

The third reason was family. Conflicts between parents such as parental discord, parents' divorce or conflict in communication between parents and children were also concerns of children.

“*My grandmother is a person who respects men and despises women, so my family often quarrels at the dinner table. I like school more but because of the covid-19 epidemic, the whole family has to stay at home due to social distancing, so almost every day I see family quarrels. I feel very tired”* (A male student in public school).

Parents' expectations for their children put students under significant pressure. “*I wanted to enter an art school but my parents expected me to take the pedagogical exam. I really didn't want that. I tried to talk to my parents but they didn't listen to me. I feel like I have to live my parents' life, not mine. They never listen to me. Mom always told me to do this and that. I'm 18 years old, but why does my mother have to decide for me? I'm so negative that I don't want to do anything, even normal things like eating, I don't want to eat. At school, I don't want to talk to anyone and I feel that my friends are also gradually shunned because of my negativity. I don't know who to share it with”* (A male student in private school).

To contain the stress all students employed a coping strategy to avoid a breakdown. From our interactions, several coping strategies for the lockdown-induced stress and anxiety were identified from the responses of the study participants. Among the ways to cope with anxiety, high school students chose the most effective way to deal with anxiety, which was “Self-motivation” such as enjoying healthy activities to reduce *anxiety* such as playing sports, journaling, watching movies, and listening to music. Some of them tried to write diaries to express their feelings.

“*I used to keep diaries, but my parents randomly rummaged through them and read them and scolded me. Now I'm sad or negative, I just cry and cry, I just want to go out and be alone until I'm okay again”* (A female student in public school).

Another solution was playing sports (“*when I feel tired, I often invite my friends to play basketball to relax*” or “*I listen to music or participate in outdoor activities*”). In addition to affirming that they could overcome anxiety (the positive side), some students responded to anxiety with negative behaviors such as “*getting into the game, the only thing I can do make yourself happy*” (A male student in public school).

The second response was “*Need to have a reasonable timetable*”. Organizing a reasonable timetable helped students reduce anxiety when the exam came. For students who did not know how to organize and used time appropriately, especially those who have difficulty adapting to difficult situations, it could cause anxiety for students.

“Parents care about feelings” was also a solution: “*For me, the best way to deal with anxiety is the care of parents for their children. Whenever I feel difficult about something, I often confide in my mother. Mom always listens and helps me overcome those difficulties.”*

The final solution was meeting school counselors. This result showed that, although high schools had psychological counseling rooms, high school students still did not know about the school counseling room, and did not know where they could meet the counselors to share their stories. That was why, even though students tried to motivate themselves to reduce anxiety, this situation had not been overcome, especially during the COVID-19 epidemic, when they had to stay at home due to social distancing and perhaps they did not know how to relieve anxiety.

## 4 Discussion

The study's results suggest that an investigation into the mental or cognitive health status of high school students was necessary to illuminate the precise psychological repercussions caused by the COVID-19 pandemic. The emergence of the COVID-19 pandemic is believed to engender intense personal stress and have consequential effects on the mental health of individuals. The coping tendencies of students in Vietnam are impacted by some factors, including symptoms of mental health conditions and social stressors related to uncertainty surrounding the COVID-19 pandemic.

Throughout history, the world has experienced several perilous epidemics, including the recent outbreak of COVID-19. It is noteworthy that the non-adoption of coping mechanisms can significantly impact the academic accomplishments of students, owing to the uncertain nature of the COVID-19 pandemic (13). The findings of this study demonstrate a significant prevalence of anxiety disorder symptoms among students during the COVID-19 lockdown period. This phenomenon has also been observed during various stages of lockdown in Vietnam. Another online survey conducted on a sample of 5,315 students between the ages of 11 and 17 in Hanoi, Vietnam revealed that approximately 7.4% of participants exhibited severe symptoms of anxiety, while 67.9% reported experiencing mild to moderate symptoms. The author posited that the COVID-19 pandemic, subsequent to the closure of schools and implementation of remote learning, exerted a detrimental influence on the psychological maturation of adolescents in middle and high school (14). A study conducted in Thailand demonstrated that students who received traditional classroom (onsite) instruction were 37.8% less likely to report moderate-to-severe anxiety in comparison to those who were engaged in fully online learning (15). Our results show higher percentages compared to other studies, such as 37.4% in China during the first year of COVID-19 (16), 36% in the second year in America (17), and 25% in June 2021 and 16% in September 2021 in Germany (18). Another internet-based study that included 212 adolescents and 662 young adults who filled out surveys in the Fall of 2019 and 2020, both before and after the COVID-19 pandemic began, suggested that rates of adolescents experiencing anxiety symptoms rose from 24.3% to 28.4% following the onset of COVID-19 (19). The lower global research results compared to our study may be attributed to several factors. Our research was conducted during the period when the Hanoi government implemented an extended period of social distancing and lockdown measures (beginning in April 2021), resulting in increased impact of COVID-19 on the economic situation of households and the health of students (20). Extended periods of social distancing, anxiety surrounding contagion, limited understanding, social stigmas, and financial burdens have been posited by researchers as factors contributing to a heightened adverse psychological effect (21). Secondly, the implementation of online learning in Hanoi during this period has not proven to be significantly effective, leading to heightened stress and anxiety among students. A study conducted in Vietnam revealed that among secondary school students, 56.7% reported a preference for online learning, and 62.7% perceived themselves to make progress through online learning (22). The corresponding proportions among high school students are 52.6% and 54.3%, respectively (22). The pressure of online learning on time management must ensure the physical and mental wellbeing of students, as well as concerns about the transition exams that students may not have been able to study for certain subjects. This is a matter of concern, impacting the holistic development of students. Furthermore, the learning activities of students in the process of online learning are quite diverse. However, not all academic activities receive appropriate attention from teachers, especially those with the unique characteristics of online learning (22). This will have a significant impact on the academic quality of students and subsequently lead to implications for students' mental health.

Vietnam implemented measures of social distancing in certain provinces and urban areas with elevated rates of COVID-19 transmission, while also sustaining the implementation of remote learning and assessments. The transition to online education and examinations has been associated with heightened levels of depression and anxiety symptoms (23). Numerous nations have opted to either suspend or delay the administration of university entrance examinations as a mitigative measure. The postponement of classes due to challenges with online learning has elicited a positive response from some students, who perceive online learning as ineffective. However, there are also opposing views, with some arguing that such a mode of study may contribute to heightened stress levels (24). A distinct schism within the online public discourse in Vietnam gives rise to inquiries regarding the encompassing nature of distance learning (25). Socioeconomically disadvantaged students encounter challenges in accessing and adhering to online classes, which results in heightened levels of anxiety and psychosocial disturbances (26).

This research has identified specific subpopulations of students that exhibit greater susceptibility to the manifestation of symptomatic indications related to both mental and physical health. In comparison to male participants, female respondents and individuals identifying as non-binary demonstrated an increased tendency to report symptoms of mental health, aligning with the observed prevalence in previous scholarly literature. A notable proportion of individuals within different demographic groups exhibit a markedly higher propensity to experience symptoms of anxiety, with a particular emphasis on those residing in urban areas and students in their twelfth year of education. The heightened levels of pressure originating from parental or peer influence may offer a plausible rationale for the observed phenomenon in comparison to their counterparts.

This study also demonstrates a significant correlation between coping mechanisms and levels of anxiety. In this study, 40.6% of students reported experiencing at risk of anxiety disorder symptoms, ranging from mild to severe levels. The evaluation of students' coping strategies entails the assessment of various factors such as their capacity to actively seek social support, the degree of isolation and mental detachment they experience, as well as their responsiveness to humanitarian concerns (27).

The present study elucidates the diverse coping mechanisms employed by students during qualitative analysis, encompassing a broad spectrum of approaches ranging from seeking social support to engaging in relaxation techniques, and in some instances, avoidance tactics. According to the findings, individuals who present symptoms of anxiety tend to resort to avoidance as a coping mechanism more frequently than they adopt alternative strategies. The present findings are congruent with the research outcomes revealed by other previous studies (28, 29). Both of these studies present findings that were obtained during a period of pronounced COVID-19 outbreak in their respective countries. Accordingly, it can be inferred that students felt compelled to utilize any feasible techniques at their disposal to mitigate their anxiety levels.

The implementation of COVID-19 policies, including social distancing, isolation, and a sedentary lifestyle, has been linked to a negative impact on the mental health of young adults (29). It has been noted in recent research that students may encounter a variety of negative emotional responses, including stress with the cancellation of exams and anticipated events, anxiety spurred by the academic workload, and fear of contracting an infection. The findings of this study indicate that an increased frequency of in-person educational activities, in the absence of interventions aimed at mitigating the root causes of psychological distress, may not yield a beneficial impact on the mental health of students.

The study has limitations that must be addressed for further exploration of the phenomenon. The survey did not differentiate between compulsory and voluntary remote education, which could introduce confounding factors associated with mental health difficulties, such as having a family member with a high medical risk or expressing reservations about the school's capacity to guarantee safety. The aforementioned factors may have exerted an influence on the selection of schooling modality by children, thereby potentially undermining the conclusiveness of the observed associations. Furthermore, it is important to acknowledge that our research utilized a convenience sampling method via the online survey, thereby imposing limitations on the extent to which the findings can be generalized to all high schools due to the non-random nature of the sample selection process. However, it is important to note that the sample size was substantial and can be considered as a strong representation of a specific group of high school students in a large urban area such as Hanoi. Finally, the GAD-7 is utilized as a screening tool rather than a diagnostic tool, which has led to our inability to assess the actual prevalence of anxiety disorders but only to evaluate the prevalence of symptoms associated with anxiety disorders. Further research is necessary to explore alternative diagnostic methods for assessing anxiety disorders within this particular demographic.

This study indicates a variety of implications for the wider community. Firstly, the administration of the educational institution should possess an understanding of the strategies utilized by students to navigate through adversities and challenges. During the period of the outbreak, it is imperative to ensure that appropriate care and assistance are provided to students who are not living with their parents or other relatives. Secondly, to effectively confront the widespread psychological difficulties faced by students, it may be advantageous for educational institutions to contemplate the implementation of interventions such as an online platform for sharing personal experiences, along with incentivizing engagement through the provision of awards or financial assistance. Thirdly, to facilitate the adoption of adaptive coping strategies and minimize the adverse impact of stress on mental well-being, it is recommended that high schools prioritize the incorporation of approach-coping techniques into their educational curricula. Furthermore, it is imperative to recognize the importance of integrating coping interventions as a fundamental element of high school and other educational programs. No research has examined the temporal evolution of mental health symptoms in relation to the length of school closures. However, it is reasonable to speculate that the detrimental impact on mental health is expected to escalate as school restrictions persist. Additional research is essential to gain a deeper understanding of this correlation.

## 5 Conclusions

The present investigation demonstrates a noteworthy level of anxiety symptoms among high school students situated in Hanoi, Vietnam amidst the global crisis of COVID-19. Furthermore, this research seeks to identify associated factors that can aid in identifying susceptible groups and formulating targeted interventions. As the global COVID-19 pandemic persists, our research outcomes may hold significant implications for analogous regions within Vietnam, as well as other countries, on addressing the mental health predicaments of secondary school learners.

## Data availability statement

The raw data supporting the conclusions of this article will be made available by the authors, without undue reservation.

## Ethics statement

The studies involving humans were approved by Institutional Review Board, University of Social Sciences and Humanities. The studies were conducted in accordance with the local legislation and institutional requirements. Written informed consent for participation in this study was provided by the participants' legal guardians/next of kin.

## Author contributions

Conceptualization: PH and NV. Formal analysis: DT and NV. Investigation: DT and NL. Methodology: PH and DT. Project administration: NL. Writing—original draft and writing—review and editing: PH, DT, NL, and NV. All authors contributed to the article and approved the submitted version.
